# Brain Activities Responding to Acupuncture at ST36 (*zusanli*) in Healthy Subjects: A Systematic Review and Meta-Analysis of Task-Based fMRI Studies

**DOI:** 10.3389/fneur.2022.930753

**Published:** 2022-07-22

**Authors:** Haoming Huang, Xiaomei Yue, Xi Huang, Wenjie Long, Shangyu Kang, Yawen Rao, Jingchun Zeng, Junling Zuo, Lin Wang, Hongjuan Li, Yeqing Wang, Shijun Qiu, Weixuan Zhao

**Affiliations:** ^1^The First Clinical Medical College, Guangzhou University of Chinese Medicine, Guangzhou, China; ^2^Department of Radiology, The First Affiliated Hospital of Guangzhou University of Chinese Medicine, Guangzhou, China; ^3^Department of Geriatrics, The First Affiliated Hospital, Guangzhou University of Chinese Medicine, Guangzhou, China; ^4^Department of Rehabilitation, The First Affiliated Hospital of Guangzhou University of Chinese Medicine, Guangzhou, China; ^5^Department of Emergency, The First Affiliated Hospital of Guangzhou University of Chinese Medicine, Guangzhou, China; ^6^The First Comprehensive Department, The Second Affiliated Hospital, Guangzhou University of Chinese Medicine (Guangdong Province Hospital of Chinese Medicine), Guangzhou, China; ^7^Department of Chinese Medicine, The First Affiliated Hospital, School of Clinical Medicine of Guangdong Pharmaceutical University, Guangzhou, China

**Keywords:** acupuncture, task-based fMRI, ST36 (zusanli), brain activation, systematic review

## Abstract

**Purpose:**

Stomach 36 (ST36, *zusanli*) is one of the important acupoints in acupuncture. Despite clinical functional magnetic resonance imaging (fMRI) studies of ST36 acupuncture, the brain activities and the neural mechanism following acupuncture at ST36 remain unclear.

**Methods:**

Literature searches were conducted on online databases, including MEDLINE, Embase, Cochrane Library, Web of Science, China National Knowledge Infrastructure, Wanfang database, WeiPu database, and China Biology Medicine, for task-based fMRI studies of acupuncture at ST36 in healthy subjects. Brain regions activated by ST36 acupuncture were systematically evaluated and subjected to seed-based *d* mapping meta-analysis. Subgroup analysis was conducted on control procedures, manual acupuncture, electrical acupuncture (EA), and acupuncture-specific activations. Meta-regression analysis was performed to explore the effects of needle retention time on brain activities following ST36 acupuncture stimulation. The activated brain regions were further decoded and mapped on large-scale functional networks to further decipher the clinical relevance of acupuncturing at ST36.

**Results:**

A total of sixteen studies, involving a total of 401 right-handed healthy participants, that satisfied the inclusion criteria were included in the present meta-analysis. Meta-analysis showed that acupuncturing on ST36 positively activates the opercular part of the right inferior frontal gyrus (IFG.R), left superior temporal gyrus (STG.L), and right median cingulate/paracingulate gyri (MCG.R) regions. Needle retention time in an acupuncture session positively correlates with the activation of the left olfactory cortex, as shown in meta-regression analysis. Subgroup analysis revealed that EA stimulation may be a source of heterogeneity in the pooled results. Functional network mappings showed that the activated areas were mapped to the auditory network and salience network. Further functional decoding analysis showed that acupuncture on ST36 was associated with pain, secondary somatosensory, sound and language processing, and mood regulation.

**Conclusion:**

Acupuncture at ST36 in healthy individuals positively activates the opercular part of IFG.R, STG.L, and MCG.R. The left olfactory cortex may exhibit positive needle retention time-dependent activities. Our findings may have clinical implications for acupuncture in analgesia, language processing, and mood disorders.

**Systematic Review Registration:**

https://inplasy.com/inplasy-2021-12-0035.

## Introduction

Acupuncture, an ancient technique in traditional Chinese medicine, has been used to treat various conditions for thousands of years and has been widely accepted as an important modality of complementary therapy in modern medicine (1). Evidence has shown that acupuncture improves cerebral circulation, relieves pain, and modulates neural function (2, 3). The *deqi* sensation is the arrival of Qi, is the special feeling and reaction of the human body after filiform needles are inserted into an acupoint, and is also the key to the effect of acupuncture. It is believed that acupuncture excites afferent nerve receptors, stimulating signals that alter the central nervous system's signal integration (1). However, it is difficult to establish the central nervous effects of acupuncture in humans due to the diversity of acupoints for various clinical settings and inadequate trials.

In recent years, there has been increasing interest in studying acupuncture-related neural activities with advanced functional neuroimaging techniques, such as functional magnetic resonance imaging (fMRI), positron emission tomography, and electroencephalography (4, 5). fMRI measures the blood oxygenation level-dependent (BOLD) signals of the brain tissue, or more specifically, the oxygen demand surge in a brain region with increased neural activities, thus localizing the activity fluctuations of the brain (6). With a task-based design, fMRI is capable of measuring the temporal effects of acupuncture and capturing the activated brain regions' responses to stimulations (7).

It is important to identify the acupuncture-stimulated brain regions, which may help to elucidate the neural mechanisms of acupuncture. Among 720 acupoints on the human body, ST36 (Stomach 36, *zusanli*) is a commonly used acupoint in animal studies (8) and clinical practices for gastric disorders, stroke, pain, sleep disturbances, and some psychological diseases (9–11). Recent evidence from animal experiments has shown the neuroanatomical basis of ST36 in driving the vagal–adrenal axis, thus modulating anti-inflammatory responses in mice (12). Research in healthy individuals demonstrated the effects of ST36 in the somatosensory and motor areas, cerebellum, and limbic system (13, 14). A meta-analysis on block-design fMRI, including both patients and healthy individuals, demonstrated that acupuncture at ST36 with *deqi* sensation exclusively activates the right hemisphere of the brain, such as the right orbital part of the inferior frontal gyrus (IFG), right median cingulate, and paracingulate gyri, right supramarginal gyrus, and pons (15). Moreover, Bai et al. reported the time-varied effects of acupuncture at ST36 in fMRI (4, 16), indicating time-dependent modes of brain activity responses to acupuncture.

Without prior clinical conditions, healthy individuals are ideal subjects to identify the specific brain activities following acupuncture. Some studies have shown increased brain activities in the insula (17, 18), bilateral primary somatosensory area and the ipsilateral cerebellum, anterior cingulate cortex, superior temporal gyrus (STG) (18–20), primary visual cortex pons, medulla regions (20), and supplementary motor area (21) when acupuncture was performed on ST36 in healthy individuals; however, other studies have yielded heterogeneous results of attenuated signals in limbic and paralimbic structures, including the amygdala, anterior hippocampus, cortices of the subgenual and retrosplenial cingulate, ventromedial pre-frontal cortex, frontal, and temporal poles (17, 22), primary somatosensory (SI) cortex, secondary somatosensory (SII) cortex (21), and precuneus (13). Despite clinical fMRI studies of ST36 acupuncture, there is yet a consensus on the temporal neural activities following acupuncture at ST36 among healthy subjects.

In the present work, we summarized the current evidence of task-based fMRI studies of acupuncture at ST36 in healthy individuals with the seed-based *d* mapping (SDM) technique and functional network mapping and decoding analysis. Subgroup analysis was conducted to assess the sources of heterogeneity between manual acupuncture (MA) and electrical acupuncture (EA). Additional subgroup analysis was also conducted on control procedures (sham acupuncture, control point acupuncture, and sensory tactile stimulation) and acupuncture-specific activations to further understand the brain activation patterns of acupuncture at ST36. Meta-regression analysis was performed to explore the potential confounding effects of needle retention time in correlation with brain activities following ST36 acupuncture stimulation. Specifically, since many studies omitted cerebellar activations, the present study focused only on the activations located in the cerebrum.

## Materials and Methods

A systematic review and meta-analysis of the neural activities of ST36 acupuncture stimulation in healthy subjects was conducted according to Preferred Reporting Items for Systematic Reviews and Meta-Analyses (PRISMA) and for Acupuncture (PRISMA-A) guidelines and published research protocol on INPLASY (INPLASY2021120035).

### Search Strategy

We searched for studies indexed in the following online databases from inception to September 2021: MEDLINE, Embase, Cochrane Library, Web of Science, China National Knowledge Infrastructure, Wanfang database, WeiPu database, and China Biology Medicine. The following terms were used for the search: (“ST 36” or “ST36” or “*zusanli*”) and (“fMRI” or “functional MRI” or “functional magnetic resonance imaging”). Titles and abstracts were independently screened by two authors, and the bibliographies of the articles were checked for additional retrievable relevant studies.

### Eligibility Criteria

Studies were selected if they met the following inclusion criteria: (1) published studies on single acupoint stimulation (c) conducted on at least 10 healthy subjects; (2) including the demographic information of the study sample; (3) conducting whole-brain analysis on task-based fMRI data; and (4) reporting the peak stimulation coordinates in standardized anatomic space, such as Talairach or Montreal Neurological Institute (MNI) space, with corresponding cluster size and statistics (voxel-wise *p*-values, *z* values, or *t* scores). Studies were excluded from the final analysis if they were (1) analyses of the region-of-interest level or (2) reviews, meta-analyses, or animal studies.

### Outcome Measurement

#### Main Outcomes

The main outcomes are significantly activated brain regions in Talairach/MNI coordinates with corresponding cluster statistics (voxel-wise *p*-values, *z* values, or *t* scores) for baseline vs. activation comparison on ST36 acupuncturing or control procedures, for an instant, sham acupuncture, control point acupuncture, and sensory tactile stimulation.

#### Secondary Outcomes

The secondary outcomes are the two-sample (or group-level) comparison among acupuncture stimulation at ST36 and control procedures (sham acupuncture, control point acupuncture, and sensory tactile stimulation) regarding significantly activated brain regions (in Talairach/MNI coordinate) with corresponding cluster statistics (voxel-wise *p*-values, *z* values, or *t* scores) between groups.

### Data Extraction

There were two authors independently extracted data from the original studies using a predesigned data extraction form. The following information was collected: first author's name, publication year, studied region, ethnicity, sex, sample size, dominant hand of the participants, experimental design, intervention, MRI acquisition protocols, neuroimaging processing software, and the coordinate system used in reporting of the results, as well as the correction method. Any discrepancies were resolved by referring to the original articles or consulting the lead author.

### Meta-Analysis Procedures

For the SDM meta-analysis, peak stimulated brain regions with cluster statistics (*p*-values or *z* values or *t* scores) were also collected. In general, the studies reported either Talairach or MNI coordinates, a text file renamed to the first author containing peak coordinates, and the associated *t* score was created. Additionally, a datasheet, including the first author's name, *t* threshold for significant clusters, number of participants in the experiment, and control groups, was created. For studies that reported multiple comparisons, the results of the acupuncture (ST36) and sham tests were recorded. For studies that reported *p*-values or *z* values, the statistics were converted to *t* scores using the online SDM converter. If a study did not provide information about the *t* threshold or corrected *p*-value, the minimum *t* score was used as a conservative estimation. Seed-based d Mapping with Permutation of Subject Images (SDM-PSI, version 6.2.2) is used in the current work to summarize the peak coordinates of brain activities in ST36 acupuncture stimulation (family-wise error-corrected followed by threshold free cluster enhancement with 1,000 permutations), and the results are thresholded to the statistical significance level of voxel-wise *p* < 0.005 with a cluster threshold of 50 voxels. An approach was adopted by conducting full analyses after iteratively leaving one of the included studies' datasets out to test the robustness of the results. Heterogeneity analyses with *I*^2^ statistics were carried out to examine the interstudy heterogeneity of individual clusters, and *I*^2^ > 30% was defined as major heterogeneity.

Subgroup analysis was performed for the control procedures, such as sham acupuncture, control point acupuncture, and sensory tactile stimulation. The effects of MA and EA stimulation were also analyzed to explore the difference in brain activation under different stimulation intensities. Additional subgroup analysis was conducted in studies that reported direct comparisons between the ST36 acupuncture and control procedures to unmask the acupuncture-specified activated brain regions.

The needle retention time was meta-regressed as a variate to study the time-dependent heterogeneity of the activation in the brain during acupuncture at ST36. In the present meta-regression, if there were multiple stimulation blocks, the scanning length of the first stimulation block was regarded as the needle retention time (min). If the study measured the brain activities for a prolonged period after one stimulation was applied and reported a series of activations with the time the activations appeared, the time (min) an activation was detected was regarded as the needle retention time. The data were extracted according to different needle retention times. For the exploratory nature of meta-regression, stringent thresholds were applied (voxel-wise *p* < 0.0005 and cluster size > 50 voxels) to decrease false-positive findings.

The included studies were placed into a non-randomized comparative design; thus, methodological index for non-randomized studies (MINORS) (23) was used to assess the methodological quality of the studies. Publication bias was assessed by graphical inspection of the asymmetry of the funnel plot for the *z* score and evaluated through Egger's test.

### Mapping on Brain Network and Functional Decoding

To decipher the meta-analysis results at the network level, the identified ST36 brain activations in the meta-analysis were mapped onto large-scale functional networks based on whole-brain functional connectivity analysis (24). To further interpret the function of the identified brain regions, we used the Neurosynth database (http://www.neurosynth.org) for data-driven functional decoding. Briefly, relevant terms for which the meta-analysis map showed the largest correlations with the identified brain regions were extracted, and terms with similar functional meanings were merged and retained the largest correlation value.

## Results

### Study Characteristics

**Figure 1:**
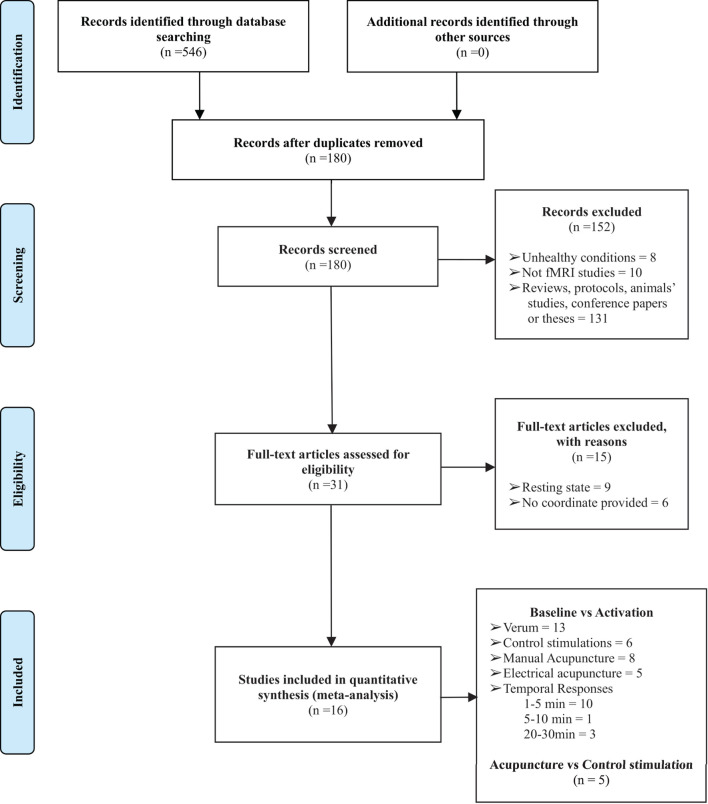
The PRISMA flow diagram of the detailed search and literature selection processes. fMRI, functional magnetic resonance imaging.

A total of 16 studies were retrieved in the preliminary literature search. All 16 studies satisfied the eligibility criteria (4, 13, 16–22, 25–31). The detailed search processes are presented in the PRISMA flowchart in Figure 1. In total, 401 right-handed healthy participants consisting of 199 male and 202 female subjects were included in the current study. The characteristics of the included studies are summarized in Table 1. A total of two included studies were conducted in the United States, 12 studies were conducted in China, 1 study was conducted in Korea, and the remaining study was conducted in Germany. Interventions included acupuncture at ST36 with *deqi* sensation with manual manipulations or electrical stimulation, whereas the control procedures included sham acupuncture, control point acupuncture, and local anesthesia before acupuncture at ST36. All the included studies involved applying different stimulations to the same group (or partially the same group) of subjects with a multi-block design or in different sessions. A total of eleven studies conducted acupuncture at the right ST36, 3 studies conducted acupuncture at the left ST36, 1 study stimulated bilateral ST36, and 1 study performed MA at the right ST36 and EA on the left ST36. Neuroimages were acquired through a 1.5 or 3.0 Tesla MRI scanner. Analysis of Functional NeuroImages (AFNI) and statistical parametric mapping (SPM) were the dominant neuroimaging processing software among the included studies, and all results were reported in Talairach and MNI coordinate systems. Studies that reported activations from different stimulation procedures were extracted separately for subsequent subgroup analysis. Finally, baseline vs. activation results from 13 studies of verum acupuncture were used to synthesize the pooled effects of acupuncture at ST36 (4, 13, 17, 18, 22, 25–31), and baseline vs. activation results from 6 studies were pooled for the control procedure effects (4, 13, 16, 17, 22, 25).

**Table 1:** The characteristics of the included studies.

**Study–author (Publication–year)**	**Conducted region**	**Ethic**	**Stimulation**	**Activation type**	**Block design**	**MRI scanner**	**TR/TE/Flipping–Angle/ Slices–Thickness/Gap–/FOV/Matrix–size**	**Acupuncture protocol**	**Control**	**Processing software**	**Coordinate system**	**Study participants**	**Age (Years)**	**Sex (M/F)**	**Handiness**	**MINORS scores**
Hui et al. (22)	America	Asian−1 Hispanic−2 White−12	MA, Sensory control	Baseline- activation	Multiple Stimulation Blocks	1.5-T Siemens Sonata	4000 ms/30 ms/90°/ 3.0 mm/0.6 mm/200 × 200 mm/64 × 64	**Location**:–right–ST36 **Needle–diameter**: −0.22 mm **Insertion–depth**:−2–3 cm **Manipulation**:–rotate–needle–in−1 HZ–for−2 min	Tap–on–right–ST36–acupoint–with–monofilament	AFNI	Talairach	15	29.8 ± 7.5	8/7	Right	19
Napadow et al. (17)	America	Caucasian−10 Hispanic−1 African=-1 American−1 Asian−1	MA, EA, Sensory control	Baseline- activation	Multiple Stimulation Blocks	3.0-T Siemens Allegra	4000 ms/30 ms/90°/ 3.0 mm/0.6 mm/200 × 200 mm/64 × 64	**Location**: MA–left–ST36 EA–right–ST36 **Needle–diameter**: −0.22 mm **Insertion–depth**:−1–1.5c **Manipulation**: MA–rotate–needle–in−1 HZ–for−1 min EA−0.7–3.6 mA–biphasic–rectangular–pulses–for−1 min (1 ms–for−2 HZ,−0.2 ms–for−100 HZ)	Tap–on–left–ST36–acupoint–with–monofilament	AFNI	Talairach	13	21–42	6/7	Right	19
Li et al. (19)	China	NA	MA, Sham	Stimulus- stimulus	Single Stimulation Block	1.5-T Siemens	3000 ms/50 ms/90°/ 6.0 mm/1.2 mm/ 220 × 220 mm/64 × 64	**Location**:–right–ST36 **Needle–diameter**: −0.30 mm **Insertion–depth**:–NA **Manipulation**:–rotate–needle–in−1 HZ–for−3 min	CP−1–located–on–lower–right–leg–below–the–knee CP−2–located–on–the–top–of–the–right–foot	SPM	Talairach	54	23.7 ± 3.6	31/24	Right	18
Bai et al. (16)	China	NA	MA, Sham	Baseline- activation	Multiple Stimulation Blocks	3.0-T GE Signa–Lx	1500 ms/30 ms/90°/ 5.0 mm/0.0 mm/240 × 240 mm/64 × 64	**Location**:–right–ST36 **Needle–diameter**: −0.20 mm **Insertion–depth**:−2–3 cm **Manipulation**:–rotate–needle–in−1 HZ–for−1 min	2–3 cm–lateral–to–right–ST36	SPM	Talairach	26	21.4 ± 1.8	13/13	Right	18
Jiang et al. (25)	China	NA	EA	Baseline- activation	Single Stimulation Block	1.5-T Marconi	3000 ms/40 ms/90°/ NA/NA/240 × 240 mm/64 × 64	**Location**:–right–ST36 **Needle–diameter**: −0.25 mm **Insertion–depth**:−3 cm **Manipulation**: −3-8V−5 HZ–continuous–EA–pulse–for−5 min	-	SPM	Talairach	16	18–28	10/6	Right	19
Jiang et al. (26)	China	NA	MA	Baseline-activation	Single Stimulation Block	1.5-T Marconi	3000 ms/40 ms/90°/ NA/NA/240 × 240 mm/64 × 64	**Location**:–right–ST36 **Needle–diameter**:−0.25 mm **Insertion–depth**:−3 cm **Manipulation**:–reinforcing–or–reducing–techniques with−3–8V−5 HZ–continuous–EA–pulse–for−5 min	-	SPM	Talairach	32	23.8 ± 4.3	18/14	Right	19
Jiang et al. (27)	China	NA	EA, Sham	Baseline-activation	Single Stimulation Block	1.5-T Marconi	3000 ms/40 ms/ 90°/ NA/NA/240 × 240 mm/64 × 64	**Location**:–right–ST36 **Needle–diameter**: −0.25 mm **Insertion–depth**:−3 cm **Manipulation**: −3–8V−5 HZ–continuous–EA–pulse–for−5 min	2–3 cm–horizontally–lateral–to–right–ST36	SPM	Talairach	13	NA	7/6	NA	19
Bai et al. (4)	China	NA	MA, Sham	Baseline-activation	Single Stimulation Block	3.0-T GE Signa–Lx	1500 ms/30 ms/ 90°/ 5.0 mm/0.0 mm/240 × 240 mm/ 64 × 64	**Location**:–right–ST36 **Needle–diameter**: −0.20 mm **Insertion–depth**:−2-3 cm **Manipulation**:–rotate–needle–in−1 HZ–for−1.5 min	2-3 cm–apart–from–right–ST36	SPM	Talairach	16	22.5 ± 1.8	8/7	Right	18
Cho et al. (20)	Korea	NA	MA, Pressure Control	Stimulus- stimulus	Multiple Stimulation Blocks	3.0-T Philips Achieva–Best	3000 ms/35 ms/ 90°/ 4.0 mm/0.0 mm/230 × 230 mm/ 64 × 64	**Location**:–left–ST36 **Needle–diameter**: −0.25 mm **Insertion–depth**:−1.5–2 cm **Manipulation**:–rotate–needle–in−2 HZ–for−0.5 min	Pressure–on–right–ST36–with–cotton–tip	SPM	Talairach	10	55–65	5/5	Right	19
Liu et al. (21)	China	Asian−18	MA, Sham	Stimulus- stimulus	Single Stimulation Block	3.0-T GE Signa–Lx	1500 ms/30 ms/ 90°/ 5.0 mm/0.0 mm/240 × 240 mm/ 64 × 64	**Location**:–right–ST36 **Needle–diameter**: −0.22 mm **Insertion–depth**:−2–3 cm **Manipulation**:–rotate–needle–in−2 HZ–for−1.5 min	2–3 cm–horizontally–lateral–to–right–ST36	SPM	Talairach	18	24.2 ± 2.9	9/9	Right	19
Hu et al. (28)	China	NA	MA	Baseline- activation	Multiple Stimulation Blocks	1.5-T GE Signa–HDxt	3000 ms/50 ms/ 90°/ 5.0 mm/0.0 mm/240 × 240 mm/ 64 × 64	**Location**:–left–ST36 **Needle–diameter**:−0.3 mm **Insertion–depth**:–NA **Manipulation**:–thrust–and–rotate–needle–in−1 HZ–for−0.5 min	-	SPM	Talairach	20	25–29	0/20	Right	18
Sun et al. (29)	China	NA	MA	Baseline- activation	Multiple Stimulation Blocks	3.0-T Siemens Allegra	2000 ms/30 ms/ 90°/ 5.0 mm/0.0 mm/240 × 240 mm/ 64 × 64	**Location**:–right–ST36 **Needle–diameter**:−0.3 mm **Insertion–depth**:−2–3 cm **Manipulation**:–rotate–needle–in−1 HZ–for−1 min	-	SPM	Talairach	50	23.3 ± 2.1	25/25	Right	19
Jin et al. (18)	China	NA	MA, local anesthesia	Baseline-activation Stimulus-stimulus	Multiple Stimulation Blocks	3.0-T Siemens	2000 ms/30 ms/ 90°/ 5.0 mm/0.0 mm/240 × 240 mm/ 64 × 64	**Location**:–right–ST36 **Needle–diameter**: −0.25 mm **Insertion–depth**:−2 cm **Manipulation**:–rotate–needle–in−1 HZ–for−1–min	Subcutaneous –(2 cm–depth)–injection–of lidocaine −2ml–(5ml:0.1g)–before–acupuncture–on–right–ST36	SPM	Talairach	40	22–25	20/20	Right	17
Li et al. (30)	China	NA	MA	Baseline-activation	Multiple Stimulation Blocks	1.5-T Siemens Symphony	4000 ms/30 ms/ 90°/ NA/NA/192 × 192 mm/ 64 × 64	**Location**:–bilateral–ST36 **Needle–diameter**:−0.25 mm **Insertion–depth**:−1.5–2.0 cm **Manipulation**: −2 HZ−2mA–for−3 min	-	-	Talairach	40	21–32	20/20	RIght	19
Nierhaus et al. (13)	German	NA	MA, CP	Baseline-activation, Stimulus- stimulus	Single Stimulation Block	3.0-T Siemens Verio	2000 ms/30 ms/ 90°/ 4.0 mm/5.0 mm/ NA/NA	**Location**:–right–ST36 **Needle–diameter**:−0.2 mm **Insertion–depth**:−1–2 cm **Manipulation**:–rotate–needle–in−1–1.5 HZ and–lift-thrusting−0.3–0.5 cm–in–depth–random–stimulation for−13–21s–within−6 min	CP–located–in–L5–dermatome between–Gallbladder–and–Bladder–meridian–skin–area–lateral–to–right–ST36	SPM	Talairach	22	22–32	11/11	Right	21
Wei et al. (31)	China	NA	MA, ultrasound stimulation	Baseline- activation	Multiple Stimulation Blocks	3.0-T Siemens Trio–Tim	3000 ms/30 ms/ 90°/ 3.0 mm/0.6 mm/200 × 200 mm/ 64 × 64	**Location**: –left–ST36 **Needle–diameter**: −0.32 mm **Insertion–depth**:−2–3 cm **Manipulation**:–rotate–needle–in−2 HZ–for−1–min	824k HZ–and−1.75w–ultrasound–stimulation–on–left–ST36	SPM AFNI	MNI	16	21–30	8/8	Right	19

*MA, manual acupuncture; EA, electric acupuncture; CP, control point; NA, not available*.

### Methodology Assessments

The potential methodological quality of the study and risk of bias were examined through the MINORS tool. Most of the studies failed to address the unbiased assessment process (blind to the evaluation of the end points, item 5), and none of the studies mentioned the sample size calculations or power estimation. Due to no current optimal standard of interventions for the neural effect of acupuncture (positive control), the included studies adopted negative control approaches, such as control point acupuncture, pressure or sensory tactile stimulation of the skin, control point acupuncture, and local anesthesia. We rated items 9 to 1 for all the studies. Hence, the overall MINORS scores ranged from 17 to 21.

### Meta-Analysis

**Figure 2:**
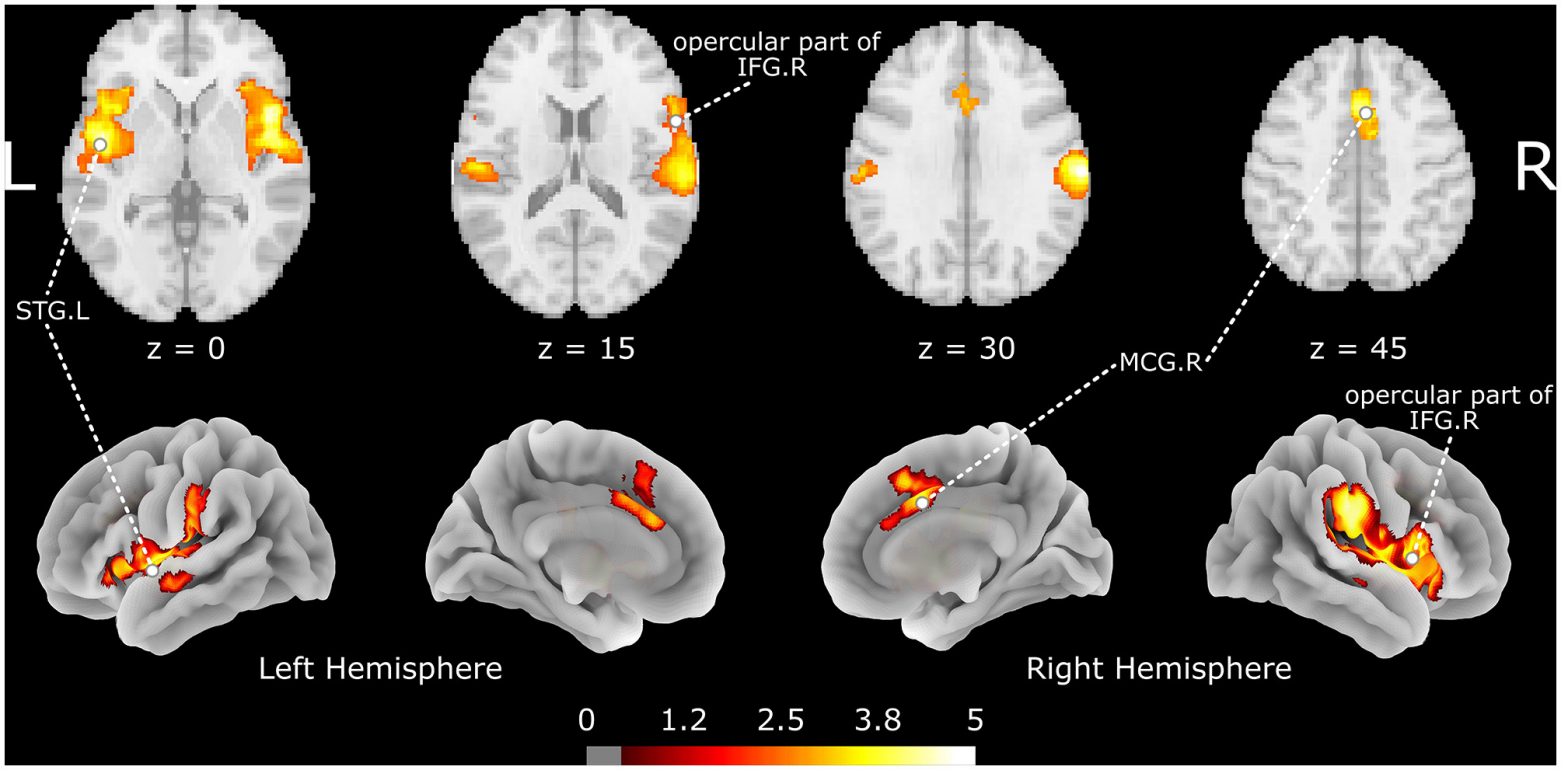
The brain regions activated by acupuncture at ST36. The color map indicates the SDM-*Z* values with a cutoff level of 0.5; STG, superior temporal gyrus; IFG, inferior frontal gyrus; MCG, median cingulate/paracingulate gyri; L/R indicates the left or right hemisphere.

The meta-analysis shows that acupuncture at ST36 positively activates three brain clusters (Figure 2 and Table 2), namely, the opercular part of right IFGr (IFG. R; Brodmann area 48; 4946 voxels; peak MNI coordinates: 48, 12, 2; peak SDM-*Z*: 5.447; *p* < 0.001; extended to right insula, Rolandic operculum, supramarginal gyrus, and STG regions), left superior temporal gyrus (STG. L; 3,095 voxels; peak MNI coordinate: −46, −6, −6; peak SDM-*Z*: 4.893; *p* < 0.001; extended to left insula, Rolandic operculum, middle temporal gyrus, and postcentral gyrus regions), and right median cingulate/paracingulate gyri (MCG. R; Brodmann area 32; 914 voxels; peak MNI coordinates: 4, 14, 44; peak SDM-*Z*: 4.570; *p* < 0.001; extended to left median cingulate/paracingulate gyri, left anterior cingulate/paracingulate gyri, and left supplementary motor area). On the other hand, control stimulation procedures positively activate brain regions similar to but smaller than those in verum acupuncture except for MCG. R (Supplementary Table S1), such as the right Rolandic operculum (Brodmann area 48; 1648 voxels; peak MNI coordinates: 54, −14, 14; peak SDM-*Z*: 4.719; *p* < 0.001), left postcentral gyrus (Brodmann area 43; 489 voxels; peak MNI coordinate: −58, −12, 32; peak SDM-*Z*: 4.139; *p* < 0.001), and right insula (Brodmann area 47; 55 voxels; peak MNI coordinates: 36, 22, 2; peak SDM-*Z*: 4.717; *p* < 0.001). No negative activation was identified in the verum acupuncture or control stimulations. Except for MCG.R (*I*^2^ = 21.607% < 30%), the heterogeneity *I*^2^ values for the remaining identified clusters were <10%, indicating no heterogeneity among the studies. The jackknife sensitivity analysis revealed that the pooled activation regions for acupuncture at ST36 were preserved, except that MCG.R failed to be identified while excluding Napadow et al.'s study (17) on EA intensity (Supplementary Table S2), indicating that the activation of these areas might be associated with electric stimulation or stimulus strength. Left- and right-side stimulation could be confounding to the analysis, and we performed additional sensitivity analysis on the main outcomes with x-axis flipped results from left ST36 stimulation studies to reduce false-negative errors from the meta-analysis. Similar outcomes were shown, indicating robustly activated regions during acupuncture at ST36 (Supplementary Table S3 and Supplementary Figure S1). The funnel plots of the identified clusters in ST36 acupuncture were roughly symmetric, and Egger's test did not detect significant publication biases (*p* < 0.05).

**Table 2:** The brain regions activated by acupuncture at ST36.

**Anatomical region**	**MNI Coordinate**	**SDM-*Z***	***P* value**	**Voxels**	**Cluster breakdown**
Right inferior frontal gyrus, opercular part (BA 48)	48, 12, 2	5.447	<0.001	4946	Right insula (BA 48), Right rolandic operculum (BA 48), Right supramarginal gyrus (BA 48), Right superior temporal gyrus (BA 48), Right insula (BA 47), Right superior temporal gyrus (BA 22)
Left superior temporal gyrus	−46, −6, −6	4.893	<0.001	3095	Left insula (BA 48), Left superior temporal gyrus (BA 48), Left rolandic operculum (BA 48), Left superior temporal gyrus (BA 22), Left middle temporal gyrus (BA 22), Left postcentral gyrus (BA 48)
Right median cingulate / paracingulate gyri (BA 32)	4, 14, 44	4.570	<0.001	914	Right median cingulate / paracingulate gyri (BA 24), Left median cingulate / paracingulate gyri (BA 24), Left anterior cingulate / paracingulate gyri (BA 24), Right median cingulate / paracingulate gyri (BA 32), Left supplementary motor area (BA 32), Left supplementary motor area (BA 8)

*MNI, Montreal Neurological Institute; SDM, Seed-based d mapping; BA, Brodmann Area*.

### Subgroup Analysis

As shown in the meta-analysis, EA might be a potential source of heterogeneity. A total of two subgroup analyses were conducted among 8 studies that adopted manual manipulation techniques (13, 16–18, 22, 28, 29, 31) and 5 studies that performed EA (17, 25–27, 30). The results (Supplementary Table S4) showed that manual manipulation techniques positively activated the right supramarginal gyrus (Brodmann area 2; 158 voxels; peak MNI coordinates: 66, −22, 32; peak SDM-*Z*: 5.841; *p* < 0.001) and opercular part of right IFG (Brodmann area 48; 113 voxels; peak MNI coordinates: 48, 12, 2; peak SDM-*Z*: 4.586; *p* = 0.003). Interestingly, EA positively activates wider areas (Supplementary Table S5), such as the left superior frontal gyrus, medial orbital (Brodmann area 11; 2679 voxels; peak MNI coordinates: −6, 42, −8; peak SDM-*Z*: 4.003; *p* < 0.001), STG. R (Brodmann area 42; 2,095 voxels; peak MNI coordinates: 60, −22, 16; peak SDM-*Z*: 4.391; *p* < 0.001), STG. L (1,167 voxels; peak MNI coordinates: −46, −4, 0; peak SDM-*Z*: 4.197; *p* < 0.001), and left anterior thalamic projections (819 voxels; peak MNI coordinates: −12, 14, 6; peak SDM-*Z*: 4.323; *p* < 0.001). The subgroup analysis results of the manual manipulation techniques and EA indicated that electric stimulation could be the major source of heterogeneity in the pooled results.

A total of five of the included studies reported group-level comparisons of verum acupuncture and control stimulations (13, 18–21); hence, an additional subgroup analysis was conducted to address another interest of the present study to identify the specified brain activations of acupuncture. Compared to control stimulations, a small area located in the right supramarginal gyrus (Brodmann area 42; 86 voxels; peak MNI coordinates: 62, −24, 18; peak SDM-*Z*: 4.714; *p* < 0.001) was activated specifically following acupuncture on ST36 (Supplementary Table S6).

### Meta-Regression Analysis

**Figure 3:**
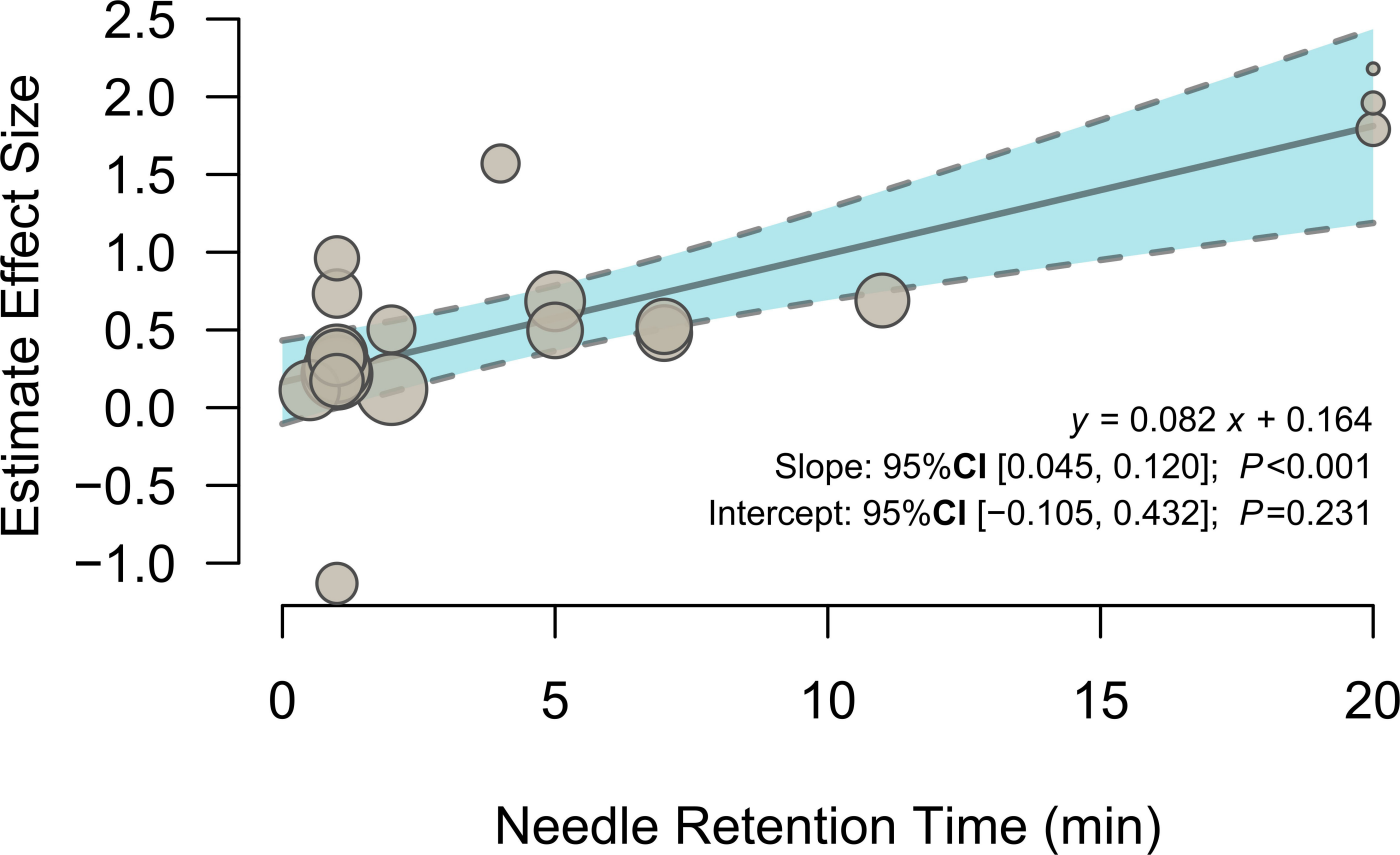
Meta-regression of the relationship between needle retention time and the estimated effect size of the left olfactory cortex.

To investigate the potential effects of needle retention time, a meta-regression analysis was conducted with the mixed-effects model. The meta-regression results (Figure 3 and Table 3) showed that activation in the left olfactory cortex (Brodmann area 42; 163 voxels; peak MNI coordinates: −4, 24, −12; peak SDM-*Z*: 3.790; *p* < 0.0005) was positively associated with needle retention time [effect size: 0.083 95% CI (0.045, 0.120), *p* < 0.001].

**Table 3:** The brain region related to needle retention time.

**Anatomical region**	**MNI Coordinate**	**SDM-*Z***	***P* value**	**Voxels**	**Cluster breakdown**
Left olfactory cortex (BA 11)	−4, 24, −12	3.790	<0.0005	163	Left gyrus rectus (BA 11), Left olfactory cortex (BA 25), Left superior frontal gyrus, medial orbital (BA 11), Corpus callosum, Left olfactory cortex (BA 11), Right superior frontal gyrus, medial orbital (BA 11)

*MNI, Montreal Neurological Institute; SDM, Seed-based d mapping; BA, Brodmann Area*.

### Large-Scale Brain Network Mapping and Functional Decoding

**Figure 4:**
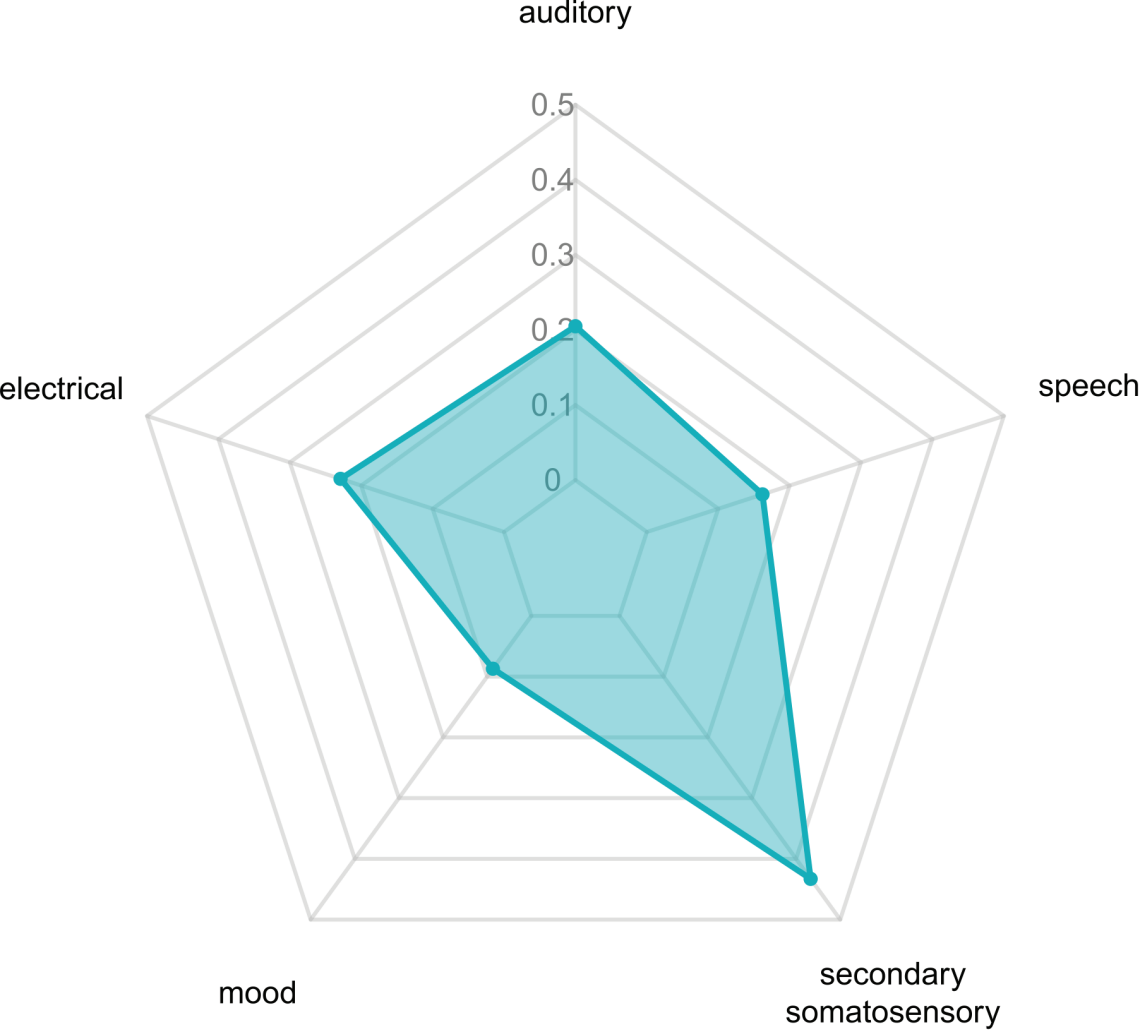
The relevant decoded functions of acupuncture at ST36.

The activated brain regions following acupuncture on ST36 were mapped on the large-scale functional network atlas, showing that the majority of voxels mapped to the auditory network, anterior salience network, and posterior salience network, and some small regions were mapped to the visuospatial network, language network, and dorsal default-mode network (DMN). To further decipher the functional effects of ST36 acupuncture, a neurosynth decoding analysis was performed. As shown in Table 4, which depicts the functional profiles, acupuncture on ST36 was associated with SII stimulation, such as pain, touch, tactile, and electrical stimulation; sound and language processing, such as auditory sensory and speech; and mood regulation (Figure 4). The full list of the decoding results is recorded in Supplementary Table S7.

**Table 4:** Brain network mapping of the brain regions activated by acupuncture at ST36.

**Network**	**Overlap voxels**	**/**	**Total network voxels**	**Percentage (%)**
Anterior salience network	966	/	4727	20.44
Auditory network	1226	/	1542	79.51
Basal ganglia network	0	/	1610	0
Dorsal DMN	12	/	7953	0.15
Higher visual network	0	/	2547	0
Language network	113	/	3850	2.94
Left ECN	0	/	4716	0
Posterior salience network	606	/	3155	19.21
Precuneus network	0	/	2635	0
Primary visual network	0	/	1120	0
Right ECN	0	/	6995	0
Sensorimotor network	0	/	5024	0
Ventral DMN	0	/	5325	0
Visuospatial network	129	/	5479	2.36
Total overlap / Total activated voxels	3052	/	8955	34.08

*DMN, default mode network; ECN, executive control network*.

## Discussion

The introduction of fMRI technology is advantageous to acupuncture research in that neural-therapeutic mechanisms of the (de)activation of brain regions under acupuncture intervention can be accessed on an image-referencing basis. Studies have shown that acupuncture of ST36 induces multiple activations of relevant brain regions, including the frontal lobe, temporal lobe, postcentral and cingulate gyri, insula, hypothalamus, hippocampus, and other limbic systems (32, 33), but the results were based on relatively small sample size and heterogeneous. The present work is a meta-analysis summarizing task-based fMRI studies of ST36 acupuncture in healthy individuals. The pooled results showed the opercular part of IFG. R, STG. L, and MCG. R regions were positively activated following ST36 acupuncturing, and no negative activations were detected. Functional network mappings of the identified clusters revealed that the activated areas were located in the auditory network, anterior salience network, and posterior salience network. Further functional decoding implied that our findings may help to elucidate the neural targets of acupuncture at ST36 for pain, speech, and mood disorder management.

Stomach 36 has been an important acupoint for improving gastrointestinal function and enhancing physical fitness since ancient China. ST36 is located in the tibialis anterior muscle four fingers beneath the lower patella margin and one finger aside from the anterior border of the tibia and is thought to strengthen the spleen and stomach, relax the meridians, activate collaterals, and strengthen the body in Chinese medicine theory. ST36 acupuncture is popular for analgesia, cognitive function and cerebral circulation improvement, and gastrointestinal disease regulation in modern practices. Studies on cerebral infarction patients and animal models have reported that acupuncture at ST36 improves brain metabolites and memory function and promotes motor function, indicating neural and cognitive recovery promoting the effects of acupuncture (34, 35). Moreover, acupuncture at ST36 has neuroprotective effects in hydrocephalus infantile rats, exhibiting anti-inflammatory and neuroprotective effects *via* inhibition of reactive astrogliosis (36).

In the present study, we mainly focused on temporal brain activities following an acupuncture session. We found that acupuncture at ST36 positively activates the opercular part of IFG. R, STG. L, and MCG. R, which is consistent with the conclusions of another meta-analysis that reported brain activations following acupuncture at ST36 with *deqi* sensation in subjects with varying background health situations (15). The clusters of the opercular part of IFG. R and STG. L were stable in jackknife analysis, indicating the robust activation of these regions during ST36 acupuncturing. The IFG and STG regions, involved in the classic Wernicke–Lichtheim–Geschwind language processing model (37), are the important components of the auditory network that are in line with the results in large-scale network mapping and functional decoding analysis. Part of the human ability to comprehend syntax and syntactic processing, as well as overall language comprehension seems to be located in the IFG (38). The STG is primarily responsible for processing sound and is also involved in processing the emotional meaning derived from facial expressions (39). The auditory network, consisting of the IFG, STG, and adjacent insula, plays an important role in language and interaction with the environment (40). However, there are limited reports on the relationships between acupuncture at ST36 and the auditory network, and the clinical applications of acupuncture in language disorders should be explored.

The activated regions also extend to bilateral insular areas that involve pain processing (41). Interestingly, it has been reported that the insular/opercular area is one of the strongest activated regions in thermal stimulation and electrostimulation and positively responds to stimulation intensity during electrostimulation (42, 43). The brain area associated with somatic nociception and central neuroendocrine system regulation is inhibited after acupuncture at ST36 (44). Multiple limbic system regions, mid-inferior occipital gyrus, precentral gyrus, and postcentral gyrus with altered long-term simultaneous activities after ST36 acupuncture associated with sensation and pain, are found in the studies (45, 46), which implies that the modes of action to the central nervous system for acupuncture analgesia may be time-dependent. Further research is needed to elucidate the short- and long-term mechanisms of acupuncture.

The MCG together with the parahippocampal gyrus are the important components of the limbic lobe with the functional of emotion processing and short- or long-term memory conversion (47). However, the cluster in the MCG was unrobust in jackknife analysis after the removal of the results from the study of Napadow *et al*. (17), which introduced EA stimulation at ST36. Although we did not identify significant heterogeneity among clusters, EA treatment could be a potential source of heterogeneity. Subgroup analyses for MA and EA stimulation were performed separately. The results revealed that the extent of activation in brain regions after EA stimulation was much greater than that after MA stimulation, indicating that different neural mechanisms may underlie EA and MA during acupuncture. A study showed that electrical stimulation at other acupoints generated different activation patterns (48), indicating that acupoint-specific activations may exist. However, Jin *et al*. (18) blocked the local sensation with lidocaine infiltration subcutaneously in healthy subjects before MA, resulting in an attenuation of the activated regions without anesthesia, indicating that cutaneous sensation (*deqi* feelings) may be the major aspect of stimulation that generates brain activation. The effects of ST36 on gastrointestinal diseases have been frequently reported (49, 50). One of the included studies showed that the oscillated secretion of gastric-related peripheral humoral factors is accompanied by hypothalamus activation in healthy subjects during EA stimulation (27). However, the pooled results and the subgroup analysis results did not show hypothalamus activity alteration, which may be due to complex gut–brain regulations that remained unclear. Additionally, subgroup analysis of control procedures revealed activation patterns similar to those following verum acupuncture. As the subgroup analysis results could not be directly compared, whether most of the acupuncture effect comes from electrical (or cutaneous) stimulations is still unknown.

We screened five studies reporting the intragroup comparison results of acupuncture vs. control for subgroup analysis to explore the ST36-specific activation of brain regions during acupuncture. The results showed that the activation of a small area located on the right supramarginal gyrus is specific to ST36 acupuncture. However, the findings conflict with the result that the regions that are specific to ST36 acupuncturing partially overlapped with the control stimulation pooled results. Moreover, based on the relationship between the group activations and the baseline, four activation modes can be observed in intragroup comparisons of task-based fMRI studies, namely, hyperactivations (treatment > control in task > baseline), hypo-activations (treatment < control in task > baseline), failures of deactivation (treatment < control in task < baseline), and hyperactivation (treatment > control in task < baseline) (51, 52), but the studies included in the subgroup analysis provide no information on the accurate activation mode, and we simply regarded the results as hyperactivation and failures of deactivation in the subgroup analysis. Thus, the result should be interpreted with caution.

Acupuncture treatment sessions generally take 5 to 40 min; hence, the relationship between needle retention time and brain activity is another important interest in the present work. Positive brain activation was found in the left olfactory cortex that positively correlated with needle retention time. Research has linked the association between acupuncture and olfactory regions. A canine model study has shown the left olfactory peduncle activation after postanesthesia acupuncture at BL60 (*Kunlun*) (13). Acupuncture at Li4 (*Hegu*) and Li20 (*Yingxiang*), as well as other acupoints, showed to improve olfactory sensitivity in healthy subjects (53) and postinfectious smell loss patients (54). Although olfactory dysfunction is one of the initial symptoms that appear years before motor symptoms and cognitive decline in several neurodegenerative diseases (55, 56), studies have tested the activation effects of acupuncture at ST36 to the left olfactory cortex on Alzheimer's animal models (57, 58), and clinical evidence on ST36 acupuncture effects in the olfactory cortex is still lacking. Our findings indicated that prolonged needle retention time during acupuncture at ST36 may strengthen the activation of the left olfactory cortex in healthy subjects, and thorough probe investigations into the clinical implications of this exploratory result may be required.

The mapping of acupuncture ST36-activated brain regions to a large-scale functional network atlas shows that most voxels map to auditory networks, the anterior salience network, and the posterior salience network, as well as the visuospatial network, language network, and dorsal DMN. The functional decoding results suggested that the activated regions were involved in pain, SII, electrical stimulation, auditory sensory and speech, and mood regulation. A study reported consistent results that acupuncture at ST36 produced the activation in the somatosensory area, insula, and the median cingulate cortex but deactivation in DMN areas (59). You *et al*. (60) reported alterations in DMN hub configurations following acupuncture at ST36. A meta-analysis of acupuncture for low back pain revealed that the brain regions involved in acupuncture were located in the salience network, DMN, pain matrix, and descending pain modulatory system (61), demonstrating the analgesic effect of acupuncture. The DMN is associated with several advanced cognitive functions; the salience network mediates the switching between the DMN and the central executive network, and those three networks are theoretically the core “triple networks” in cognition. The modulator interactions between the “triple networks” following acupuncture at ST36 need further investigation.

Notably, no SI area activation was observed in our pooled results, which may be inconsistent with some previous acupuncture fMRI results. Activations in SI have been reported in the included acupuncture studies (13, 22). However, another included study demonstrated decreased activities in the SI and SII, which might be due to ST36 inhibited or modulated pain sensory for analgesic effects (21). A recent study showed the activations in SI and SII, insula, and thalamus region during sponge scrubbing, whereas the SI region did show activation during real acupuncture at Li4 (Hegu) (62). Another study reported evidence of functional diversity within SI that higher SI activation was found in the anticipation phase (a visual cue to inform participants that pain would be delivered) and lower activation in pain stimulation, indicating the differences in attentional and sensory pain processing (63). In our results, SII activation was observed and agreed with the frequently reported results, indicating that acupuncture may involve complex sensory processing. Nonetheless, the somatosensory and analgesic mechanism for acupuncture at ST36 remains to be further researched.

The present study had the following limitations. First, the number of included studies and the total number of involved participants were relatively small. Second, the present meta-analysis did not study the effects of *deqi* sensations which are believed to be a key factor in the therapeutic effect of acupuncture. Although the acupuncture sensation scale (64), the Massachusetts General Hospital acupuncture sensation scale (65), and the Southampton needle sensation questionnaire (66) were introduced and validated, inconsistencies in the evaluation of *deqi* sensations were found across the included studies, and it was difficult to precisely define the intensities of *deqi* sensations in the present meta-analysis. Universal tools for quantifying the *deqi* phenomenon may be of value in further research. Third, there is a relatively large publication year span of the included studies. Techniques and software have been substantially updated, which may introduce inestimable heterogeneity to the results. Forth, despite in most experimental paradigms, the needles were inserted into the skin before the scan and stayed in place throughout the scan session, the effects of the needle penetrating the skin were ignored. The needle retention time for the meta-regression was only considered as one stimulation period or plus the following non-resting state period but not the full length of the session. Specific experimental paradigms and analysis workflow considering for the nature of the acupuncture therapeutic effects may be needed in further studies. Fifth, the included studies tested different stimulations on the same subjects, and the design inevitably faces the concerns of selection bias and incomplete washout periods. A randomized controlled design with a larger sample size should be adopted in future research. Sixth, the MINORS scores of the included studies were below the recommended cutoff for comparison studies, suggesting methodological defects in the studies. However, considering the specialized designs, developing a new assessment tool for comprehensive methodological evaluation of task-based fMRI studies may be a solution. Finally, this study examined acupuncture-specific brain activation. Because of the relatively small sample size of included studies and unclarified mode of activation in the two-sample comparisons, the results need to be interpreted with caution. Nonetheless, acupuncture-specific brain activity is an important topic that warrants rigorous clinical studies and experimental validations.

## Conclusion

Acupuncture at ST36 in healthy individuals mainly activates three clusters located in the opercular part of IFG.R, STG.L, and MCG.R. EA stimulation may expand the activated brain regions. The needle retention time during an acupuncture session may enhance the activation of the left olfactory cortex. Our findings facilitate an understanding of the mechanisms of acupuncture treatment and provide neurological evidence for the clinical implications of acupuncture in analgesia, language processing, and mood disorders.

## Data Availability Statement

The original contributions presented in the study are included in the article/Supplementary Material, further inquiries can be directed to the corresponding authors.

## Author Contributions

HH, XY, and XH performed data analyses and wrote the main manuscript. SK, YR, and YW were involved in the search and selection of eligible articles. WL, LW, and JZ extracted the data. JZ and HL were responsible for image preparation. SQ and WZ designed the research study. All authors reviewed and approved the manuscript.

## Funding

The authors disclosed receipt of the following financial support for the research, authorship, and/or publication of this article. This research was supported by the Key International Cooperation Project of National Natural Science Foundation of China (Grant No. 81920108019), the Youth Project of Guangdong Medical Research Foundation (Grant No. B2022108), the Research Foundation for Advanced Doctoral Talents of the First Affiliated Hospital/School of Clinical Medicine of Guangdong Pharmaceutical University (Grant No. KYQDJF202010), and the Project of Traditional Chinese Medicine Bureau of Guangdong Province (Grant No. 20221225).

## Conflict of Interest

The authors declare that the research was conducted in the absence of any commercial or financial relationships that could be construed as a potential conflict of interest.

## Publisher's Note

All claims expressed in this article are solely those of the authors and do not necessarily represent those of their affiliated organizations, or those of the publisher, the editors and the reviewers. Any product that may be evaluated in this article, or claim that may be made by its manufacturer, is not guaranteed or endorsed by the publisher.

## Supplementary Material

Supplementary Figure S1 The brain regions activated by acupuncture at ST36 with flipped results from left ST36 stimulation studies. The color map indicates the SDM-*Z* values with a cutoff level of 0.5; L/R indicate the left or right hemisphere.

Supplementary Table S1 The brain regions activated by control stimulation. MNI, Montreal Neurological Institute. SDM, seed-based *d* mapping; BA, Brodmann area.

Supplementary Table S2 The jackknife sensitivity analysis of the activated regions for ST36 acupuncture. BA, Brodmann area.

Supplementary Table S3 The brain regions activated by acupuncture at ST36 with flipped results from left ST36 stimulation studies. MNI, Montreal Neurological Institute; SDM, seed-based *d* mapping; BA, Brodmann area.

Supplementary Table S4 The brain regions activated by manual acupuncture at ST36. MNI, Montreal Neurological Institute; SDM, seed-based *d* mapping; BA, Brodmann area.

Supplementary Table S5 The brain regions activated by electrical acupuncture at ST36. MNI, Montreal Neurological Institute; SDM, seed-based *d* mapping; BA, Brodmann area.

Supplementary Table S6 The brain region activated is specific to verum acupuncture at ST36. MNI, Montreal Neurological Institute; SDM, seed-based *d* mapping; BA, Brodmann area.

Supplementary Table S7 Functional decoding of activated brain regions following acupuncture at ST36.

## Abbreviations

fMRI: functional magnetic resonance imaging
BOLD: blood oxygenation level-dependent
ST36: Stomach 36, zusanli
STG: superior temporal gyrus
SI: primary somatosensory
SII: secondary somatosensory
SDM: seed-based d Mapping
MA: manual acupuncture
EA: electrical acupuncture
PRISMA: Preferred Reporting Items for Systematic Reviews and Meta-Analyses
MNI: Montreal Neurologic Institute
MINORS: methodological index for non-randomized studies
IFG: inferior frontal gyrus
MCG: median cingulate/paracingulate gyri
DMN: default-mode network.
