# Supplementary figures and images for: Brain Activities Responding to Acupuncture at ST36 (zusanli) in Healthy Subjects: A Systematic Review and Meta-Analysis of Task-Based fMRI Studies
Source: Front Neurol. 2022 Jul 22;13:930753. doi: 10.3389/fneur.2022.930753 (PMC9373901; doi:10.3389/fneur.2022.930753)

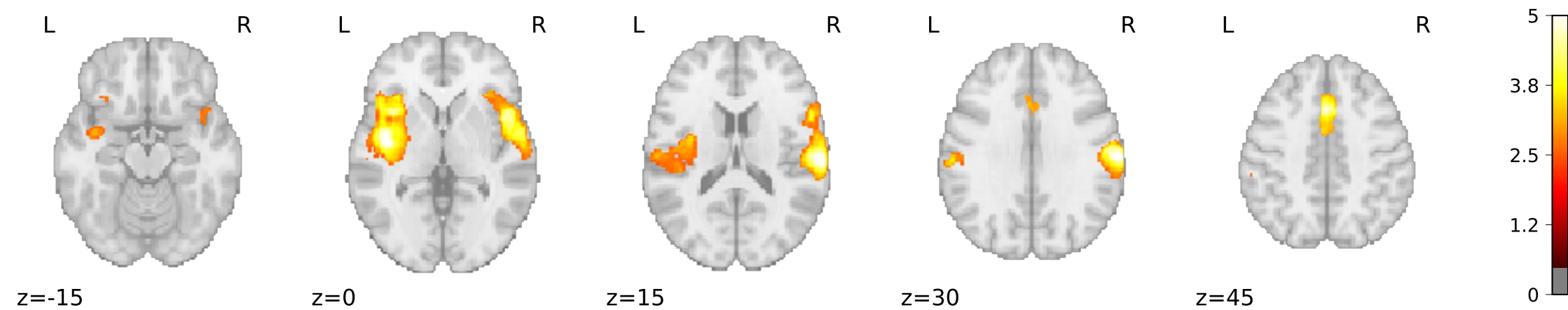

Supplement: Supplementary Figure S1 — The brain regions activated by acupuncture at ST36 with flipped results from left ST36 stimulation studies. The color map indicates the SDM-Z values with a cutoff level of 0.5; L/R indicate the left or right hemisphere. [file Image_1.pdf]
