# Supplementary material for: Brain Activities Responding to Acupuncture at ST36 (zusanli) in Healthy Subjects: A Systematic Review and Meta-Analysis of Task-Based fMRI Studies
Source: Front Neurol. 2022 Jul 22;13:930753. doi: 10.3389/fneur.2022.930753 (PMC9373901; doi:10.3389/fneur.2022.930753)
Supplement: Supplementary Table S1 — The brain regions activated by control stimulation. MNI, Montreal Neurological Institute. SDM, seed-based d mapping; BA, Brodmann area. [file Table_1.docx]

**Table S1. The brain regions activated by control stimulation.**

| Anatomical Region | MNI  Coordinate | SDM-*Z* | *P*  value | Voxels | Cluster Breakdown |
| --- | --- | --- | --- | --- | --- |
| Right rolandic operculum (BA 48) | 54, -14, 14 | 4.719 | < 0.001 | 1648 | Right rolandic operculum (BA 48), Right supramarginal gyrus (BA 48), Right superior temporal gyrus (BA 22), Right superior temporal gyrus (BA 48), Right superior temporal gyrus (BA 42), Right supramarginal gyrus (BA 2) |
|  |  |  |  |  |  |
| Left postcentral gyrus (BA 43) | -58, -12, 32 | 4.139 | < 0.001 | 489 | Left supramarginal gyrus (BA 48), Left postcentral gyrus (BA 43), Left postcentral gyrus (BA 3), Left superior longitudinal fasciculus III, Left supramarginal gyrus (BA 2), Left superior temporal gyrus (BA 48) |
|  |  |  |  |  |  |
| Right insula (BA 47) | 36, 22, 2 | 4.717 | < 0.001 | 55 | Right insula (BA 47), Right insula (BA 48) |

MNI, Montreal Neurological Institute. SDM, Seed-based d Mapping; BA, Brodmann Area.
