# Supplementary material for: Brain Activities Responding to Acupuncture at ST36 (zusanli) in Healthy Subjects: A Systematic Review and Meta-Analysis of Task-Based fMRI Studies
Source: Front Neurol. 2022 Jul 22;13:930753. doi: 10.3389/fneur.2022.930753 (PMC9373901; doi:10.3389/fneur.2022.930753)
Supplement: Supplementary Table S2 — The jackknife sensitivity analysis of the activated regions for ST36 acupuncture. BA, Brodmann area. [file Table_2.docx]

**Table S2. The jackknife sensitivity analysis of the activated regions for ST36 acupuncture.**

| Study  (Publication year) | Right inferior frontal gyrus, opercular part  (BA 48) | Left superior temporal gyrus | Right median cingulate / paracingulate gyri  (BA 32) | Additional regions |
| --- | --- | --- | --- | --- |
| Hui  (2005) | **√** | **√** | **√** | Right thalamus  Left anterior thalamic projections |
| Napadow  (2005) | **√** | **√** | **×** | - |
| Bai  (2009) | **√** | **√** | **√** | - |
| Jiang1  (2010) | **√** | **√** | **√** | - |
| Jiang2  (2010) | **√** | **√** | **√** | - |
| Jiang3  (2010) | **√** | **√** | **√** | - |
| Bai  (2010) | **√** | **√** | **√** | - |
| Hu  (2012) | **√** | **√** | **√** | Left paracentral lobule  (BA 4) |
| Sun  (2012) | **√** | **√** | **√** | - |
| Jin  (2014) | **√** | **√** | **√** | - |
| Li  (2014) | **√** | **√** | **√** | - |
| Nierhaus  (2015) | **√** | **√** | **√** | - |
| Wei  (2020) | **√** | **√** | **√** | - |

BA, Brodmann area.
