# Supplementary material for: Brain Activities Responding to Acupuncture at ST36 (zusanli) in Healthy Subjects: A Systematic Review and Meta-Analysis of Task-Based fMRI Studies
Source: Front Neurol. 2022 Jul 22;13:930753. doi: 10.3389/fneur.2022.930753 (PMC9373901; doi:10.3389/fneur.2022.930753)
Supplement: Supplementary Table S3 — The brain regions activated by acupuncture at ST36 with flipped results from left ST36 stimulation studies. MNI, Montreal Neurological Institute; SDM, seed-based d mapping; BA, Brodmann area. [file Table_3.docx]

**Table S3. The brain regions activated by acupuncture at ST36 with flipped results from left ST36 stimulation studies.**

| Anatomical Region | MNI  Coordinate | SDM-*Z* | *P*  value | Voxels | Cluster Breakdown |
| --- | --- | --- | --- | --- | --- |
| Right supramarginal gyrus (BA 2) | 66, -22, 32 | 5.512 | < 0.001 | 2984 | Right rolandic operculum (BA 48), Right insula (BA 48), Right supramarginal gyrus (BA 48), Right superior temporal gyrus (BA 48), Right superior temporal gyrus (BA 22), Right postcentral gyrus (BA 43), Right supramarginal gyrus (BA 2) |
|  |  |  |  |  |  |
| Left rolandic operculum | -44, -8, 4 | 5.383 | < 0.001 | 2897 | Left insula (BA 48), Left superior temporal gyrus (BA 48), Left rolandic operculum (BA 48), Corpus callosum  Left heschl gyrus (BA 48), Left lenticular nucleus, putamen (BA 48), Left insula (BA 47), Left inferior frontal gyrus, orbital part (BA 47) |
|  |  |  |  |  |  |
| Left supplementary motor area (BA 32) | -2, 14, 44 | 4.890 | < 0.001 | 243 | Left median cingulate / paracingulate gyri (BA 24), Left supplementary motor area (BA 32), Right median cingulate / paracingulate gyri (BA 24), Left supplementary motor area (BA 8), Right median cingulate / paracingulate gyri (BA 32), Left median cingulate / paracingulate gyri (BA 32) |

MNI, Montreal Neurological Institute. SDM, Seed-based d Mapping; BA, Brodmann Area.
