# Supplementary material for: Brain Activities Responding to Acupuncture at ST36 (zusanli) in Healthy Subjects: A Systematic Review and Meta-Analysis of Task-Based fMRI Studies
Source: Front Neurol. 2022 Jul 22;13:930753. doi: 10.3389/fneur.2022.930753 (PMC9373901; doi:10.3389/fneur.2022.930753)
Supplement: Supplementary Table S4 — The brain regions activated by manual acupuncture at ST36. MNI, Montreal Neurological Institute; SDM, seed-based d mapping; BA, Brodmann area. [file Table_4.docx]

**Table S4. The brain regions activated by manual acupuncture at ST36.**

| Anatomical Region | MNI  Coordinate | SDM-*Z* | *P*  value | Voxels | Cluster Breakdown |
| --- | --- | --- | --- | --- | --- |
| Right supramarginal gyrus (BA 2) | 66, -22, 32 | 5.841 | < 0.001 | 158 | Right supramarginal gyrus (BA 2), Right supramarginal gyrus (BA 48), Right postcentral gyrus (BA 43), Right supramarginal gyrus (BA 1), Right supramarginal gyrus (BA 43) |
|  |  |  |  |  |  |
| Right inferior frontal gyrus, opercular part (BA 48) | 48, 12, 2 | 4.586 | 0.003 | 113 | Right inferior frontal gyrus, opercular part (BA 48), Right insula (BA 48), Right inferior frontal gyrus, opercular part (BA 45), Right inferior frontal gyrus, triangular part (BA 48), Right inferior frontal gyrus, opercular part (BA 44) |

MNI, Montreal Neurological Institute; SDM, Seed-based d Mapping; BA, Brodmann Area.
