# Supplementary material for: Brain Activities Responding to Acupuncture at ST36 (zusanli) in Healthy Subjects: A Systematic Review and Meta-Analysis of Task-Based fMRI Studies
Source: Front Neurol. 2022 Jul 22;13:930753. doi: 10.3389/fneur.2022.930753 (PMC9373901; doi:10.3389/fneur.2022.930753)
Supplement: Supplementary Table S5 — The brain regions activated by electrical acupuncture at ST36. MNI, Montreal Neurological Institute; SDM, seed-based d mapping; BA, Brodmann area. [file Table_5.docx]

**Table S5. The brain regions activated by electrical acupuncture at ST36.**

| Anatomical Region | MNI  Coordinate | SDM-*Z* | *P*  value | Voxels | Cluster Breakdown |
| --- | --- | --- | --- | --- | --- |
| Left superior frontal gyrus, medial orbital (BA 11) | -6, 42, -8 | 4.003 | < 0.001 | 2679 | Corpus callosum, Left gyrus rectus (BA 11), Right gyrus rectus (BA 11), Left superior frontal gyrus, medial orbital (BA 11), Right superior frontal gyrus, medial orbital (BA 11), Left superior frontal gyrus, medial (BA 10) |
|  |  |  |  |  |  |
| Right superior temporal gyrus (BA 42) | 60, -22, 16 | 4.391 | < 0.001 | 2095 | Right rolandic operculum (BA 48), Right superior temporal gyrus (BA 22), Right superior temporal gyrus (BA 48), Corpus callosum, Right supramarginal gyrus (BA 48), Right superior temporal gyrus (BA 42) |
|  |  |  |  |  |  |
| Left superior temporal gyrus | -46, -4, 0 | 4.197 | < 0.001 | 1167 | Left superior temporal gyrus (BA 48), Left insula (BA 48), Left rolandic operculum (BA 48), Corpus callosum, Left heschl gyrus (BA 48), |
|  |  |  |  |  |  |
| Left anterior thalamic projections | -12, 14, 6 | 4.323 | < 0.001 | 819 | Left anterior thalamic projections, Left caudate nucleus, Right anterior thalamic projections, Left caudate nucleus (BA 25), Right thalamus, Left thalamus |

MNI, Montreal Neurological Institute; SDM, Seed-based d Mapping; BA, Brodmann Area.
