# Supplementary material for: Brain Activities Responding to Acupuncture at ST36 (zusanli) in Healthy Subjects: A Systematic Review and Meta-Analysis of Task-Based fMRI Studies
Source: Front Neurol. 2022 Jul 22;13:930753. doi: 10.3389/fneur.2022.930753 (PMC9373901; doi:10.3389/fneur.2022.930753)
Supplement: Supplementary Table S6 — The brain region activated is specific to verum acupuncture at ST36. MNI, Montreal Neurological Institute; SDM, seed-based d mapping; BA, Brodmann area. [file Table_6.docx]

**Table S6.** **The brain region activated is specific to verum acupuncture at ST36.**

| Anatomical Region | MNI  Coordinate | SDM-*Z* | *P*  value | Voxels | Cluster Breakdown |
| --- | --- | --- | --- | --- | --- |
| Right supramarginal gyrus, BA 42 | 62, -24, 18 | 4.714 | < 0.001 | 86 | Right supramarginal gyrus (BA 48), Right superior temporal gyrus (BA 42), Right supramarginal gyrus (BA 42), Right rolandic operculum (BA 48), Right superior temporal gyrus (BA 48), Right supramarginal gyrus (BA 2) |

MNI, Montreal Neurological Institute; SDM, Seed-based *d* Mapping; BA, Brodmann Area.
