# Supplementary material for: Brain Activities Responding to Acupuncture at ST36 (zusanli) in Healthy Subjects: A Systematic Review and Meta-Analysis of Task-Based fMRI Studies
Source: Front Neurol. 2022 Jul 22;13:930753. doi: 10.3389/fneur.2022.930753 (PMC9373901; doi:10.3389/fneur.2022.930753)
Supplement: Supplementary Table S7 — Functional decoding of activated brain regions following acupuncture at ST36. [file Table_7.docx]

| **Physical / Psychological term** | **Correlation** |
| --- | --- |
| pain | 0.433 |
| painful | 0.427 |
| secondary somatosensory | 0.355 |
| somatosensory | 0.322 |
| tactile | 0.254 |
| noxious | 0.242 |
| stimulation | 0.229 |
| electrical | 0.229 |
| touch | 0.224 |
| sounds | 0.21 |
| music | 0.206 |
| auditory | 0.205 |
| pitch | 0.201 |
| acoustic | 0.199 |
| listening | 0.178 |
| nociceptive | 0.17 |
| intensity | 0.167 |
| speech | 0.162 |
| noise | 0.153 |
| discriminative | 0.149 |
| opercular | 0.144 |
| sensation | 0.14 |
| auditory stimuli | 0.131 |
| speech perception | 0.129 |
| empathy | 0.125 |
| spoken | 0.123 |
| heard | 0.103 |
| suffering | 0.1 |
| conflict | 0.099 |
| inhibition | 0.099 |
| response inhibition | 0.095 |
| mood | 0.087 |

**Table S7. Functional decoding of activated brain regions following acupuncture at ST36.**
